# Nonsteroidal Anti-inflammatory Drugs as Prophylaxis for Heterotopic Ossification after Total Hip Arthroplasty: A Systematic Review and Meta-Analysis

**DOI:** 10.1097/MD.0000000000000828

**Published:** 2015-05-08

**Authors:** Shun-Li Kan, Bo Yang, Guang-Zhi Ning, Ling-Xiao Chen, Yu-Lin Li, Shi-Jie Gao, Xing-Yu Chen, Jing-Cheng Sun, Shi-Qing Feng

**Affiliations:** From the Department of Orthopaedics, Tianjin Medical University General Hospital, Heping District, Tianjin, China.

## Abstract

Heterotopic ossification (HO) is a frequent complication after total hip arthroplasty (THA). Nonsteroidal anti-inflammatory drugs (NSAIDs) have been used as routine prophylaxis for HO after THA. However, the efficacy of NSAIDs on HO, particularly selective NSAIDs versus nonselective NSAIDs, is uncertain.

We searched PubMed, Embase, the Cochrane Central Register of Controlled Trials, and clinicaltrials.gov to identify randomized controlled trials with respect to HO after THA. Two reviewers extracted the data and estimated the risk of bias. For the ordered data, we followed the Bayesian framework to calculate the odds ratio (OR) with a 95% credible interval (CrI). For the dichotomous data, the OR and 95% confidence interval (CI) were calculated using Stata version 12.0. The subgroup analyses and the Grading of Recommendations, Assessment, Development and Evaluation (GRADE) approach were used.

A total of 1856 articles were identified, and 21 studies (5995 patients) were included. In the NSAIDs versus placebo analysis, NSAIDs could decrease the incidence of HO, according to the Brooker scale (OR = 2.786, 95% CrI 1.879–3.993) and Delee scale (OR = 9.987, 95% CrI 5.592–16.17). In the selective NSAIDs versus nonselective NSAIDs analysis, there was no significant difference (OR = 0.7989, 95% CrI 0.5506–1.125) in the prevention of HO. NSAIDs could increase discontinuation caused by gastrointestinal side effects (DGSE) (OR = 1.28, 95% CI 1.00–1.63, *P* = 0.046) more than a placebo. Selective NSAIDs could decrease DGSE (OR = 0.48, 95% CI 0.24–0.97, *P* = 0.042) compared with the nonselective NSAIDs. There was no significant difference with respect to discontinuation caused by nongastrointestinal side effects (DNGSE) in NSAIDs versus a placebo (OR = 1.16, 95% CI 0.88–1.53, *P* = 0.297) and in selective NSAIDs versus nonselective NSAIDs (OR = 0.83, 95% CI 0.50–1.37, *P* = 0.462).

NSAIDs might reduce the incidence of HO and increase DGSE in the short-term.

## INTRODUCTION

Total hip arthroplasty (THA) is an effective treatment for severe pain and handicap induced by hip joint disease.^1,2^ Heterotopic ossification (HO) commonly follows THA as a frequent complication,^3^ occurring in 16% to 53% of the patients undergoing THA.^4^ HO might induce a reduced range of hip motion and severe pain.^5,6^ Therefore, decreasing the occurrence of HO for patients undergoing THA has been considered.

Irradiation after surgery could decrease the incidence of HO.^7,8^ However, high costs and the risk of soft tissue sarcoma inhibit the use of irradiation. Increased trials have demonstrated that nonsteroidal anti-inflammatory drugs (NSAIDs) are effective for the prevention of HO.^9–12^ Therefore, NSAIDs have been widely used for the prophylaxis of HO. However, the risk of gastrointestinal side effects caused by NSAIDs has drawn the attention of surgeons. Several studies showed that NSAIDs had a favorable influence on the prophylaxis of HO after THA, whereas other studies demonstrated that NSAIDs did not provide efficient prevention against the risk of HO.^13^ A previous Cochrane review^14^ investigated NSAIDs as prophylaxis for HO; however, that review had several defects. It included 2 quasi-randomized trials, applied an imprecise statistical analysis method, investigated NSAIDs versus a placebo rather than selective NSAIDs versus nonselective NSAIDs, and did not estimate the quality of evidence of every involved article. A recent meta-analysis^15^ investigated the difference between selective NSAIDs and nonselective NSAIDs as prophylaxis for HO after THA. This meta-analysis used an inexact statistical analysis method and did not evaluate the risk of bias and the quality of evidence of each involved article.

Because of the shortcomings of previous meta-analyses, their conclusions were not robust. Hence, we executed a Bayesian meta-analysis of randomized controlled trials to evaluate the efficacy and safety of NSAIDs for the prevention of HO after THA.

## MATERIALS AND METHODS

### Search Strategy

We searched for the published results with respect to HO after THA in PubMed, Embase, the Cochrane Central Register of Controlled Trials, and clinicaltrials.gov. For this article, we searched databases by using the following terms (“Arthroplasty OR Replacement OR Hip” OR “arthropl∗ OR pros∗ OR surg∗ OR replac∗[tiab]” OR “hip prosthesis OR joint prosthesis”) AND “heterotopic ossification” AND (“pain [mesh]” OR “pain∗”). The initial search was limited to human subjects and RCTs regardless of the language of publication. In addition, we manually checked the reference lists of articles obtained and those from previously published systematic reviews and meta-analyses to identify the relevant reports. Ethical approval of this study was not necessary, as systematic review and meta-analyses do not involve patients.

### Inclusion and Exclusion Criteria

An article qualifying for this meta-analysis fulfilled the following inclusion criteria: participants assigned to undergo THA; treatment comparing NSAIDs versus the control or comparing two NSAID drugs; information assembled at the follow-up regarding HO, discontinuation caused by nongastrointestinal side effects (DNGSE) and discontinuation caused by gastrointestinal side effects (DGSE); and randomized controlled trials.

Trials were excluded if they were abstracts, letters, case reports, case series, reviews, guidelines, nonhuman studies, or meeting proceedings concerning our investigation question; the studies used reduplicative data; or the patients had a definite contraindication for the treatment with an NSAID (eg, a previous serious adverse reaction to an NSAID, previous major gastrointestinal bleeding, serious renal impairment, or a known bleeding disorder).

### Data Extraction and Outcome Measures

Two reviewers independently extracted the following data from the qualified publication: the first author, year of publication, geographical location, span of the surgery, type and dose of the intervention and comparison, duration of the treatment, outcomes, duration of the follow-up, patient characteristics, number of patients, and study type. For information that could not been extracted from the involved studies, communication was made with the first author and study sponsors to obtain the relevant material. In the case of a disagreement not solved by discussion, a third reviewer judged the study.

The primary outcome measure for this meta-analysis was radiographically determined HO. In the initial trials, there were several measurement scales, such as the Brooker scale, DeLee scale, Rosendahl scale, Hierton scale, and Hoikka scale, to grade the level of HO. If a certain classification method was used in only one trial, we described it in the narrative form. If the measurements of HO were made multiple times, the last outcome was recorded. The main secondary outcome measures were DNGSE and DGSE.

### Risk of Bias Assessment

Risk of bias assessment of each involved article was conducted in light of the Cochrane Handbook for Systematic Reviews of Interventions (version 5.1.0),^16^ using Review Manager, version5.3 (The Nordic Cochrane Centre, The Cochrane Collaboration, Copenhagen, 2014). The content for the assessment consisted of the sequence generation (selection bias), allocation sequence concealment (selection bias), blinding of the participants and personnel (performance bias), blinding of the outcome assessment (detection bias), incomplete outcome data (attrition bias), selective outcome reporting (reporting bias), and other biases including whether the study was supported by external funding and whether the baseline was balanced. The judgment for each entry involved assessing the risk of bias as “low risk,” as “high risk,” or as “unclear risk,” which indicated a lack of information or uncertainty over the potential for bias. Two reviewers independently assessed each RCT, and any disagreements were resolved by discussion and consensus.

### Quality of Evidence Assessment

We used the Grading of Recommendations, Assessment, Development and Evaluation (GRADE) approach^17^ to estimate the quality of the evidence. GRADE is a framework for illustrating the level of evidence. In this approach, the assessment was conducted based on 5 factors, including the risk of bias, inconsistency, indirectness, imprecision, and publication bias. We used GRADE Pro, version 3.6, to estimate the quality of evidence as high, moderate, low, or very low.

### Statistical Analysis

For the ordered outcome (the overall incidence of HO), we implemented the Bayesian random-effects meta-analysis according to the Bayesian framework model presented by Whitehead et al.^18^ We followed the Bayesian framework to explore the posterior distribution of HO. Noninformative priors were used to generate the outcomes. We estimated the odds ratio (OR) and 95% credible interval (CrI) by WinBUGS, version 1.4.3 (MRC Biostatistics Unit, Cambridge, UK). For the stratified model, in regard to HO, we operated 10,000 iterations and ran the burn-in of 1000 iterations. DNGSE and DGSE were conducted as the dichotomous outcomes, using Stata, version 12.0 (Stata Corp., College Station, TX). We calculated the pooled Peto OR estimates and 95% confidence interval (CI). The I^2^ test^19^ and chi-square test were used to assess the heterogeneity. When I^2^ > 50%, it indicated massive diversity between the studies, and a random-effect model was used. We used a funnel plot and Egger linear regression test to estimate the publication bias if the number of studies was larger than 10.^20^ Statistical significance was considered when the *P* value was less than 0.05. We executed the subgroup analyses by sample size and drug category to determine whether different sample sizes had effects on the estimates of DNGSE and DGSE and whether various drug categories yielded different influences on DNGSE and DGSE. We calculated their statistical power by using a commercially available software package (Power and Precision V4, Biostat). For the outcomes, a confidence level of 5% with a statistical power of 80% was used and regarded as being acceptable for medical purposes.^21^

## RESULTS

### Summary of Enrolled Studies

**FIGURE 1:**
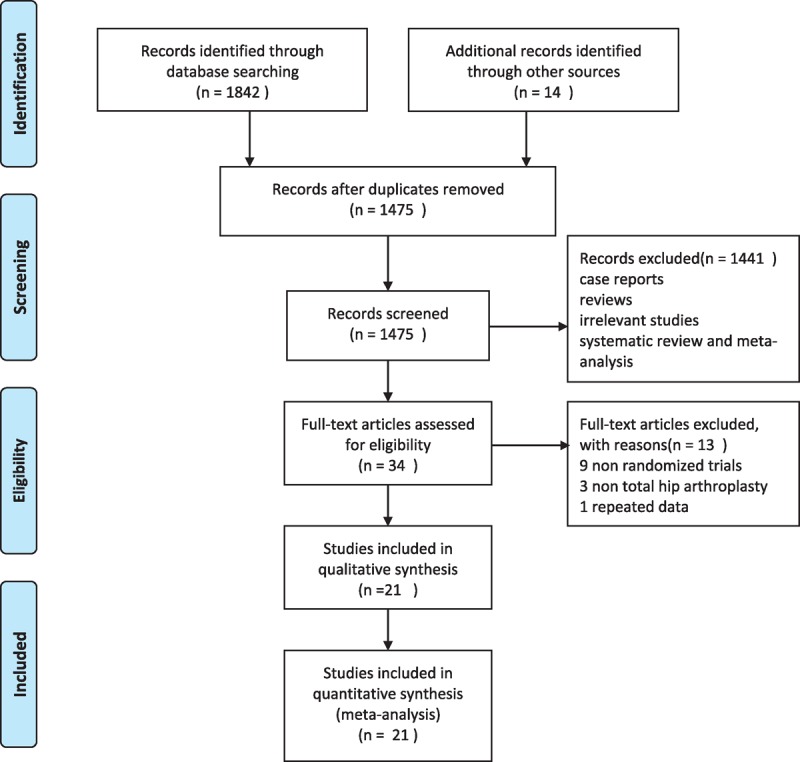
Flow chart of study selection.

We identified 1856 articles from PubMed, Embase, Cochrane Central Register of Controlled Trials, and clinicaltrials.gov, and the reference lists of the articles obtained (including those from previously published systematic reviews). Of these articles, 381 were removed due to duplicate reportage, and 1441 were excluded based on the titles and abstracts, which were case reports, reviews, irrelevant studies, systematic reviews, and meta-analyses. The remaining 34 articles were accessed for the full text and screened for further assessment, 9 of which were excluded because they were not randomized controlled trials.^22–30^ Three of the studies were excluded because they were not studies on THA.^31–33^ One article was excluded because of repeated data.^34^ Finally, 21 articles fulfilling our inclusion criteria were included in our meta-analysis, with a total of 5995 participants. Figure 1 demonstrates the flow of the inclusion process.

### Characteristics of the Studies Included in the Review

All of the included studies in our meta-analysis were randomized controlled trials. These studies were implemented from 1985 to 2011. The populations in the studies varied from 43^35^ to 2649.^13^ Two articles were published in the 1980, 12 in the 1990, and 7 in the 21st Century. A total of 17 articles were from Europe, specifically, 2 from Finland, 4 from Denmark, 4 from Sweden, 3 from German, 1 from Belgium, 1 from France, 1 from The Netherlands, and 1 from Switzerland. Two trials were conducted in North America and Asia. Two studies originated in Oceania. One study was reported in Chinese, and the other studies were in English. Detailed information regarding the involved trials is shown in Table 1.

**TABLE 1:**
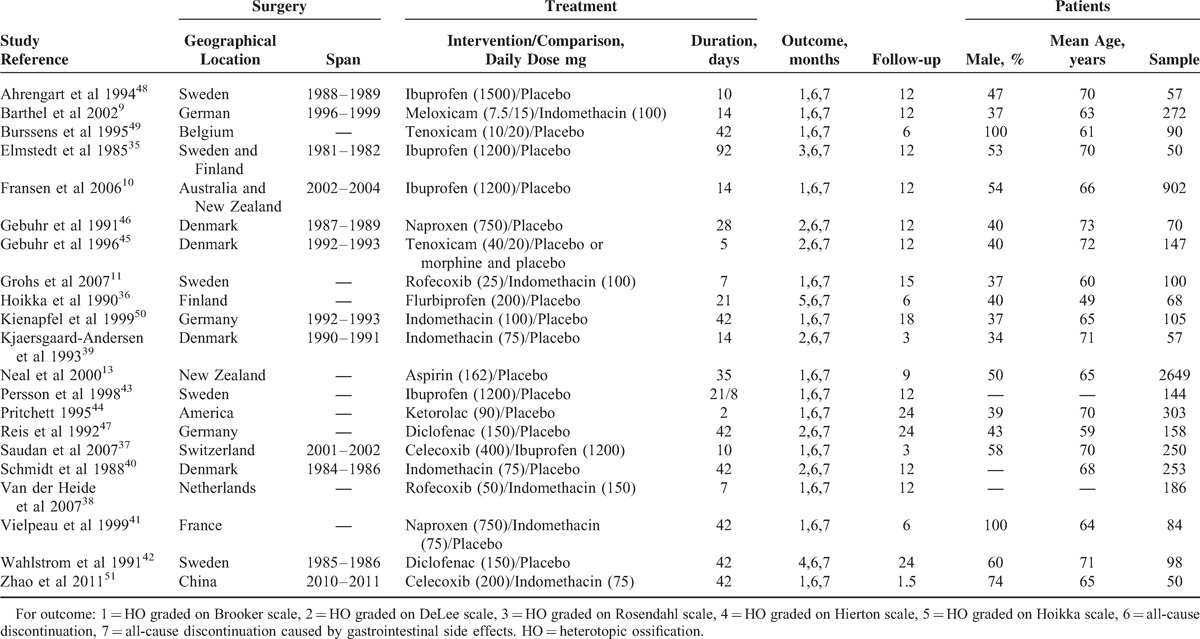
Baseline Characteristics of Studies Included in the Survey

### Risk of Bias in the Included Studies

**FIGURE 2:**
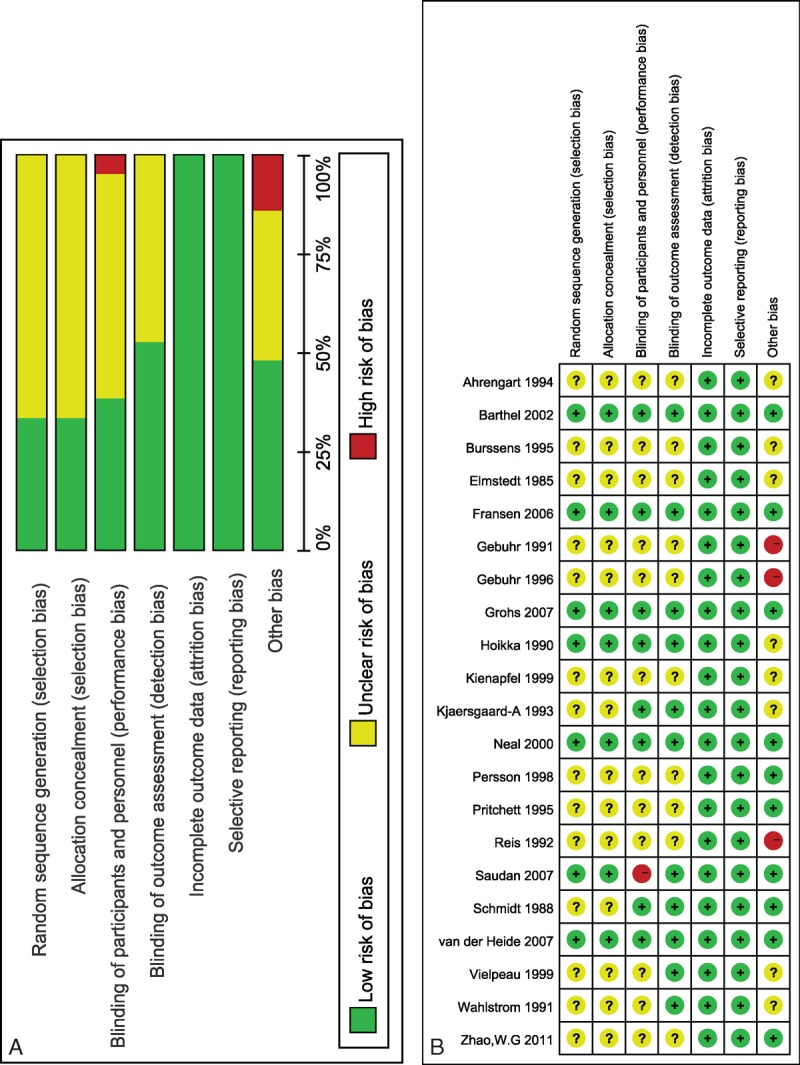
Risk of bias assessment of each included study. (A) Risk of bias graph. (B) Risk of bias summary.

We used the risk of bias tool implemented in Review Manager 5.3 to evaluate the risk of bias in light of the Cochrane Handbook for Systematic Reviews of Interventions. The particular information of the risk of bias of the included articles is demonstrated in Figure 2. All of the included articles were described as randomized. However, only seven^9–11,13,36–38^ of studies comprehensively described the generation of a randomized sequence, and the remaining studies did not demonstrate the randomization method. Blinding of participants and personnel was performed in 8 studies.^9–11,13,36,38–40^ The patients and healthcare teams were not blinded to treatment allocation in 1 study.^37^ There was insufficient information to permit judgment of “low risk” or “high risk” for the other studies. The outcome assessors did not have knowledge of the allocated interventions in 11 studies.^9–11,13,36–42^ All of the included articles displayed a low risk of bias for the incomplete outcomes and selective outcome reporting. Nine articles^9–11,13,37,38,40,43,44^ in the included studies displayed a low risk of bias for other biases, and three^45–47^ of the involved studies had a high risk of bias, with the remaining studies being indistinct.

## OUTCOMES

### NSAIDs Versus a Placebo

#### Overall Incidence of HO

Sixteen trials reported NSAIDs versus a placebo as prophylaxis against HO after THA. There were two classification methods (the Brooker scale and the Delee scale) including more than 1 article. Based on the Brooker^12^ scale, there were 8 studies on NSAIDs as prophylaxis for HO after THA. NSAIDs showed a significant difference (OR = 2.786, 95% CrI 1.879–3.993) in the prevention of HO compared with a placebo. A total of 5 studies related to NSAIDs versus a placebo contributed to the assessment of the Delee^52^ scale. Similarly, the NSAIDs significantly decreased the occurrence of HO after THA (OR = 9.987, 95% CrI 5.592–16.17) compared with a placebo.

Based on the Rosendahl, Hierton, and Hoikka scales, 3 articles were included.^35,36,42^ The 3 articles demonstrated that NSAIDs were more effective in preventing HO after THA than a placebo.

#### DNGSE

**FIGURE 3:**
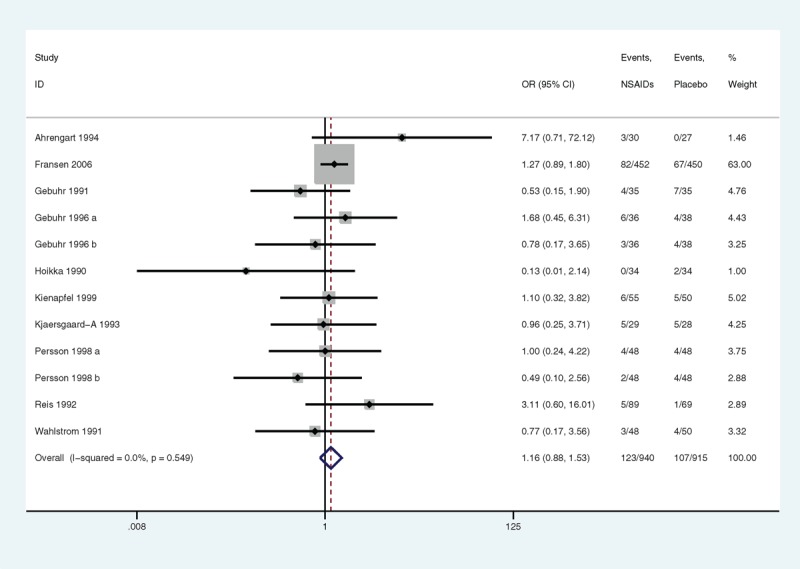
A forest plot of discontinuation caused by nongastrointestinal side effects: nonsteroidal anti-inflammatory drugs (NSAIDs) versus placebo.

**FIGURE 4:**
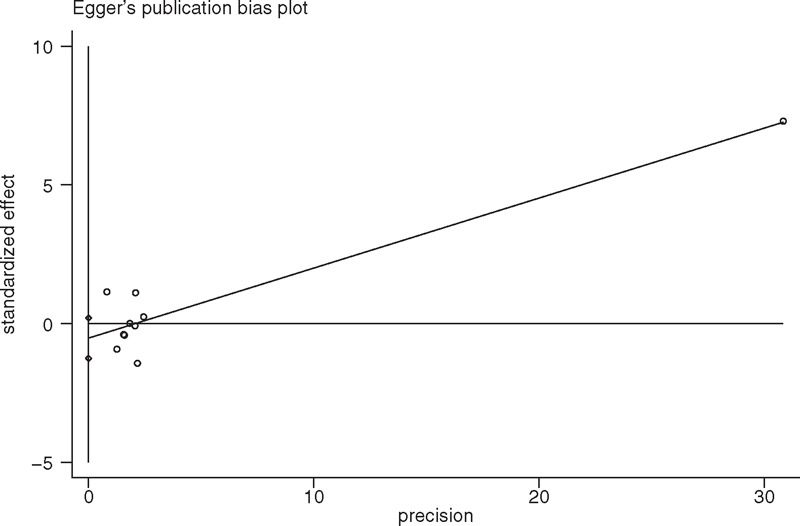
A funnel plot of discontinuation caused by nongastrointestinal side effects of the included studies comparing nonsteroidal anti-inflammatory drugs (NSAIDs) with placebo.

**FIGURE 5:**
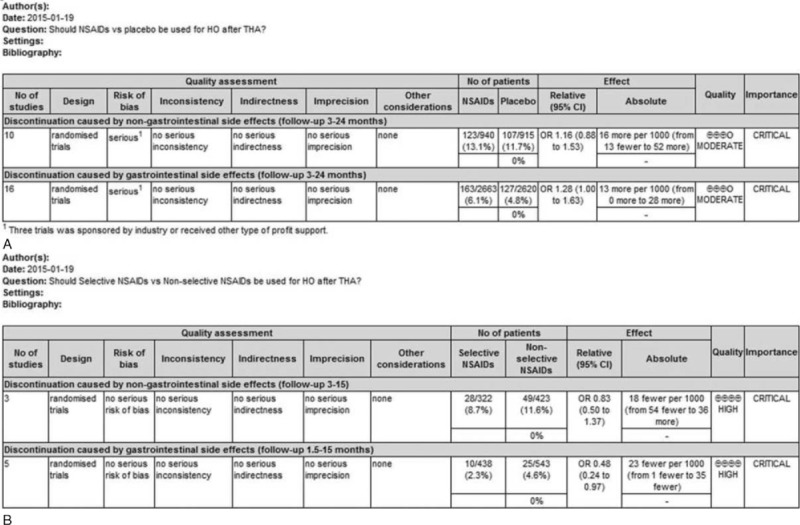
Analysis and quality of the evidence using Grading of Recommendations, Assessment, Development and Evaluation (GRADE).

Ten articles provided data on DNGSE. NSAIDs were not associated with a significant risk of DNGSE compared with a placebo. The OR was 1.16 (95% CI 0.88 to 1.53, *P* = 0.297) (Figure 3); however, statistical significance was not found, which was possibly because of insufficient power (statistical power = 10%) caused by a limited number of articles. No heterogeneity was observed (*P* = 0.549, I^2^ = 0.0%). There was no publication bias found (Egger linear regression test: *P* = 0.325) (Figure 4). The GRADE quality of the evidence was judged to be moderate (Figure 5A).

**FIGURE 6:**
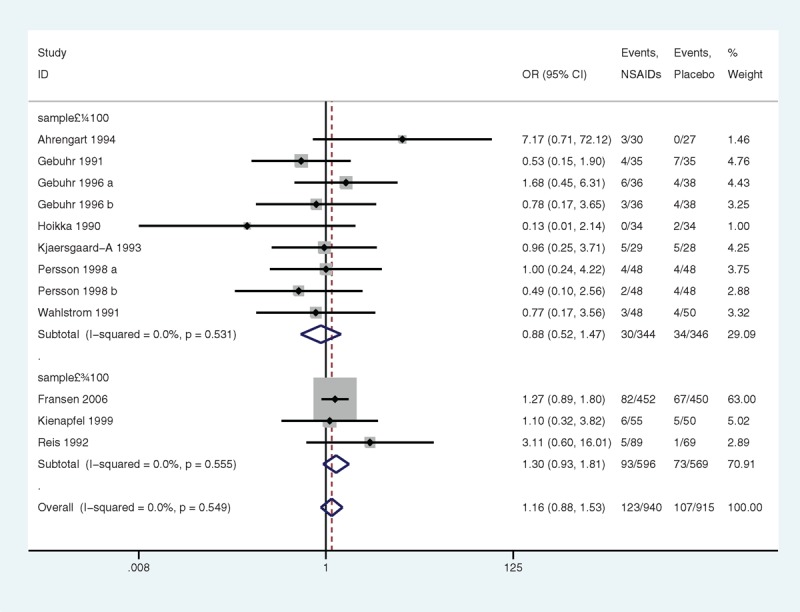
A forest plot of discontinuation caused by nongastrointestinal side effects by subgroup analysis of sample size: nonsteroidal anti-inflammatory drugs (NSAIDs) versus placebo.

**FIGURE 7:**
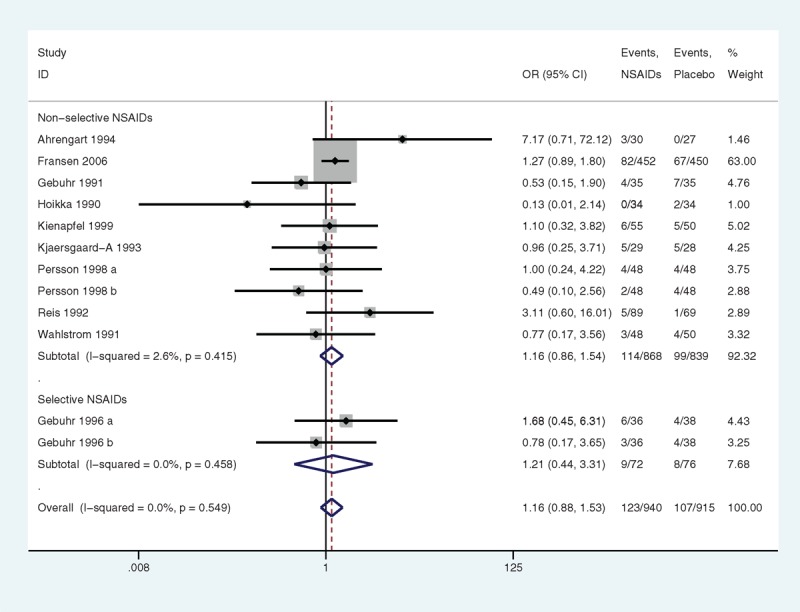
A forest plot of discontinuation caused by nongastrointestinal side effects by subgroup analysis of drug categories: nonsteroidal anti-inflammatory drugs (NSAIDs) versus placebo.

Subgroup analyses with stratification by the sample size were performed. The heterogeneity in the articles with less than 100 samples was low (*P* = 0.531, I^2^ = 0.0%) (Figure 6), and the heterogeneity in the articles with more than 100 samples was low (*P* = 0.555, I^2^ = 0.0%) as well. No significant association was indicated (*P* = 0.623 and *P* = 0.120, respectively). In the subgroup analyses relating to drug categories, the heterogeneity in nonselective NSAIDs was low (*P* = 0.415, I^2^ = 2.6%) (Figure 7), and the heterogeneity in selective NSAIDs was low (*P* = 0.458, I^2^ = 0.0%). Nonselective NSAIDs and selective NSAIDs demonstrated no significant association (*P* = 0.329 and *P* = 0.708, respectively).

#### DGSE

**FIGURE 8:**
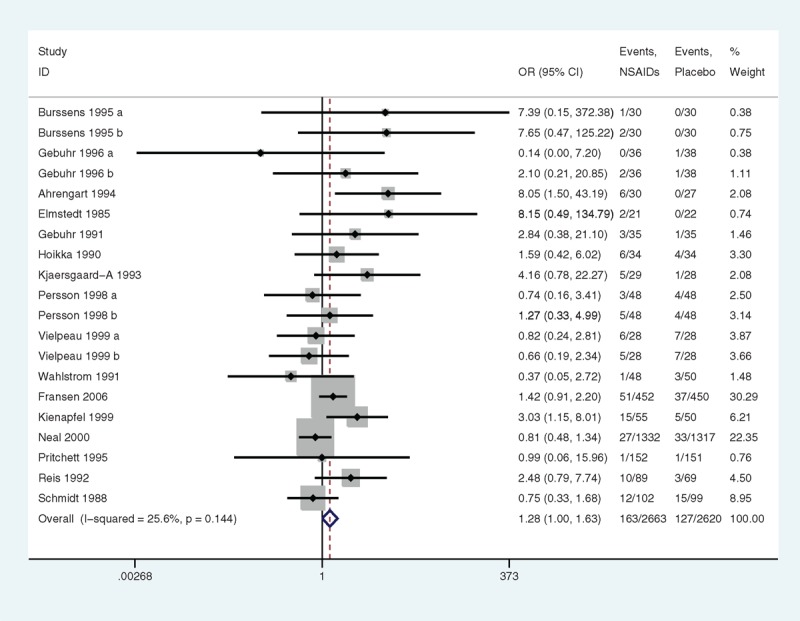
A forest plot of discontinuation caused by gastrointestinal side effects: nonsteroidal anti-inflammatory drugs (NSAIDs) versus placebo.

**FIGURE 9:**
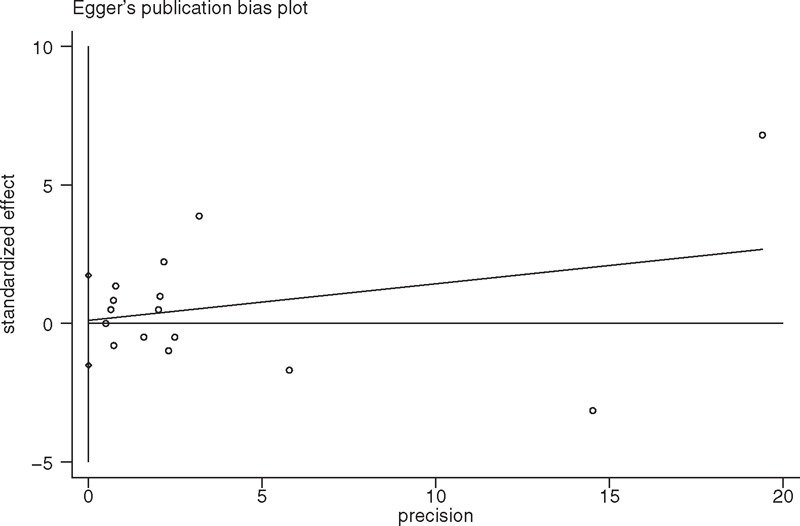
A funnel plot of discontinuation caused by gastrointestinal side effects of the included studies comparing nonsteroidal anti-inflammatory drugs (NSAIDs) with placebo.

There were 16 studies reporting on DGSE. Overall, the occurrence of DGSE among patients administered NSAIDs was 28% higher (95% CI 0%–63% higher, *P* = 0.046) (Figure 8) than DGSE among the patients assigned a placebo. The statistical power was 35.5%. No heterogeneity was observed (*P* = 0.144; I^2^ = 25.6%). No evidence of publication bias was found by Egger linear regression test (*P* = 0.885) (Figure 9). The GRADE quality of evidence was judged to be moderate (Figure 5A).

**FIGURE 10:**
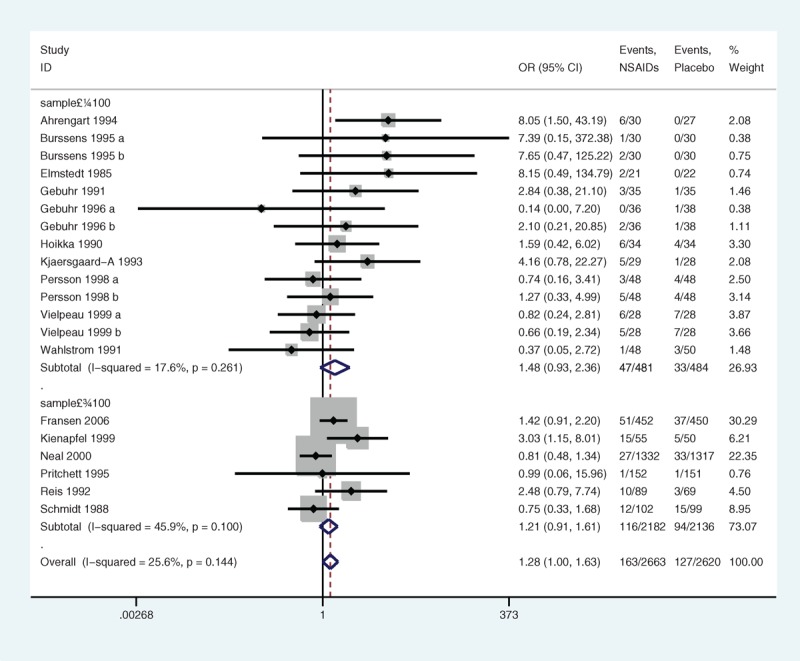
A forest plot of discontinuation caused by gastrointestinal side effects by subgroup analysis of sample size: nonsteroidal anti-inflammatory drugs (NSAIDs) versus placebo.

**FIGURE 11:**
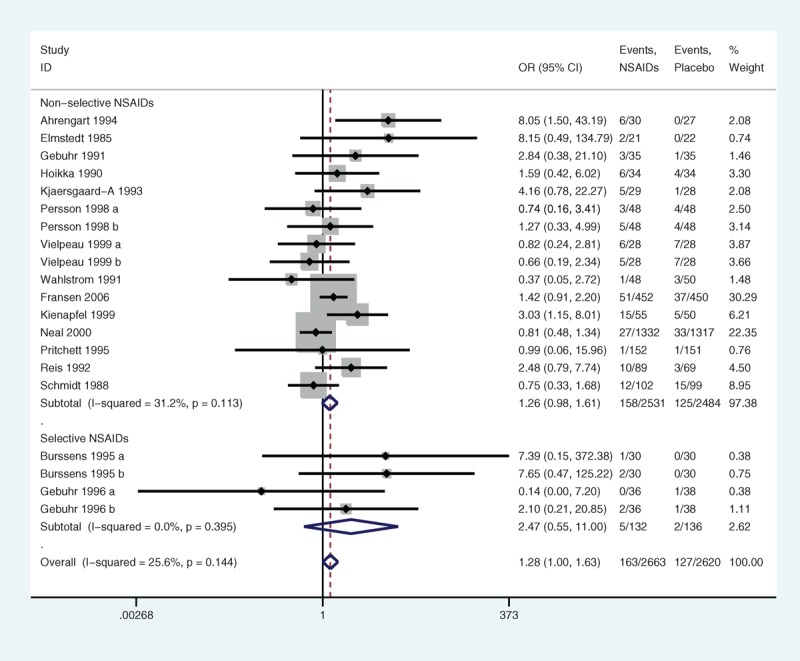
A forest plot of discontinuation caused by gastrointestinal side effects by subgroup analysis of drug categories: nonsteroidal anti-inflammatory drugs (NSAIDs) versus placebo.

The subgroup analyses stratified by sample size showed that the heterogeneity in the articles with less than 100 samples (*P* = 0.261, I^2^ = 17.6%) (Figure 10) and in the articles with more than 100 samples (*P* = 0.100, I^2^ = 45.9%) was low. There was no significant association (*P* = 0.100 and 0.183, respectively). In the subgroup of drug categories, the trials that assigned selective NSAIDs had low heterogeneity (*P* = 0.395, I^2^ = 0.0%) (Figure 11), and the trials that allocated nonselective NSAIDs had low heterogeneity (*P* = 0.113, I^2^ = 31.2%) as well. No significant association was found between selective NSAIDs and DGSE (*P* = 0.236) and between nonselective NSAIDs and DGSE (*P* = 0.068).

### Selective NSAIDs Versus Nonselective NSAIDs

#### Overall Incidence of HO

Five studies were in the group of selective NSAIDs versus nonselective NSAIDs. However, in 1 study^37^ in Sudan using the Brooker scale, the second and the third level of the Brooker scale were combined. Therefore, the Bayesian framework could not be used in this study. The other 4 eligible articles were assessed. There was no significant difference (OR = 0.7989, 95% CrI 0.5506–1.125) between the selective NSAIDs and nonselective NSAIDs as prophylaxis for HO after THA.

#### DNGSE

**FIGURE 12:**
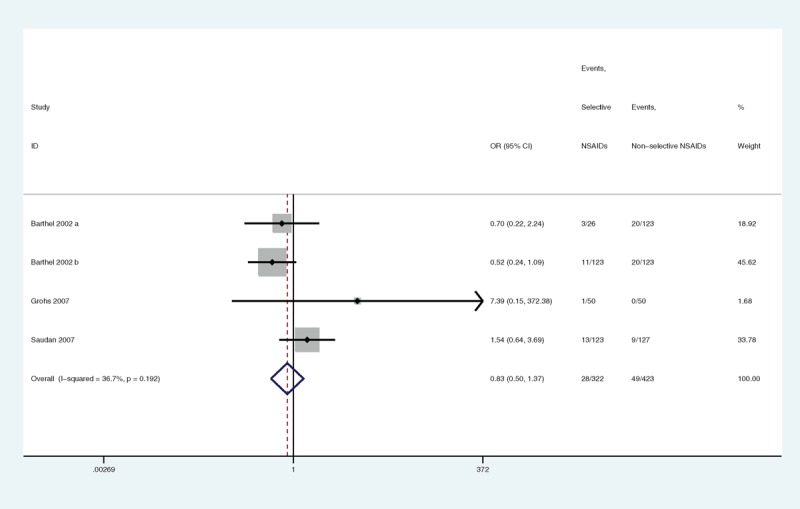
A forest plot of discontinuation caused by nongastrointestinal side effects: selective nonsteroidal anti-inflammatory drugs (NSAIDs) versus nonselective NSAIDs.

Three articles reported data on DNGSE. Selective NSAIDs did not show a significant difference (OR = 0.83, 95% CI 0.50–1.37, *P* = 0.462) (Figure 12) compared with nonselective NSAIDs. Insufficient statistical power (power = 26%) might have contributed to the effect, because the data on selective versus nonselective NSAIDs were derived from a finite number of studies. No heterogeneity was observed (*P* = 0.192, I^2^ = 36.7%). The overall GRADE quality of evidence was judged to be high (Figure 5B).

Because there were no articles with less than 100 samples, subgroup analyses stratified by sample size could not be conducted.

#### DGSE

**FIGURE 13:**
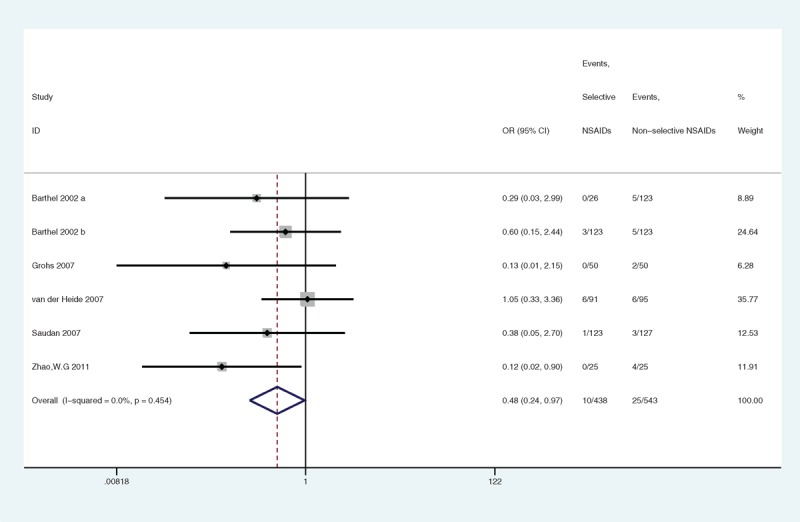
A forest plot of discontinuation caused by gastrointestinal side effects: selective nonsteroidal anti-inflammatory drugs (NSAIDs) versus nonselective NSAIDs.

There were 5 studies providing data on DGSE. Compared with nonselective NSAIDs, selective NSAIDs significantly decreased DGSE (OR = 0.48, 95% CI 0.24–0.97, *P* = 0.042) (Figure 13). The statistical power was 72%. No heterogeneity was observed (*P* = 0.454, I^2^ = 0.0%). The overall GRADE quality of evidence was judged to be high (Figure 5B).

**FIGURE 14:**
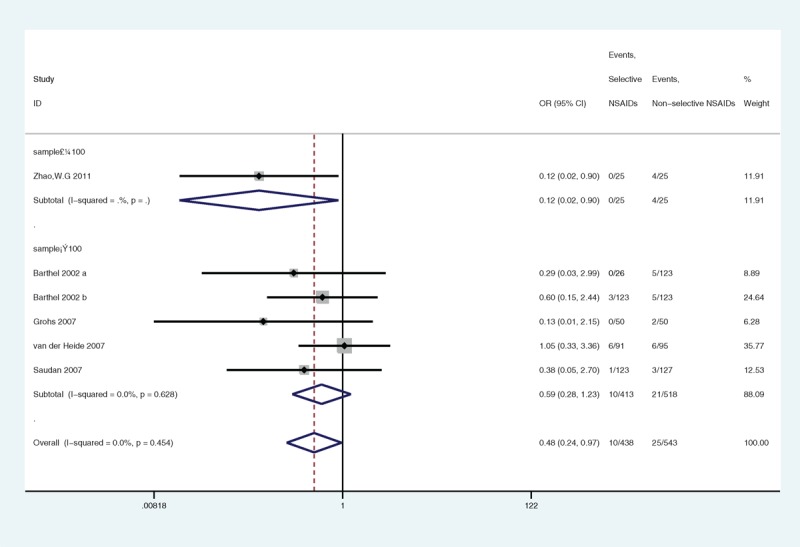
A forest plot of discontinuation caused by gastrointestinal side effects by subgroup analysis of sample size: selective nonsteroidal anti-inflammatory drugs (NSAIDs) versus nonselective NSAIDs.

In the subgroup analyses with respect to sample size, the heterogeneity in the articles with equal to or more than 100 samples was low (*P* = 0.628, I^2^ = 0.0%) (Figure 14), and no significant association was indicated (*P* = 0.159). There was 1 article with less than 100 samples, and the heterogeneity could not be calculated.

## DISCUSSION

### NSAIDs Versus a Placebo

The differences in 2 (overall incidence of HO and DGSE) of the 3 (overall incidence of HO, DNGSE, and DGSE) analyzed variables were revealed in this meta-analysis of randomized controlled trials comparing NSAIDs with a placebo. According to the Brooker scale, NSAIDs could significantly reduce the incidence of HO after THA compared with a placebo. Regarding the Delee scale, NSAIDs could significantly decrease the incidence of HO after THA in comparison with a placebo. NSAIDs were not associated with DNGSE, whereas they increased the DGSE.

The overall incidence of HO^10,35,36,39–47,48–50^ and DGSE^10,47,48^ has been reported in individual trials or meta-analyses;^14,53,54^ however, they have drawn little attention. The American Academy of Orthopaedic Surgeons emphasizes preventing venous thromboembolic disease^55^ and diagnosing periprosthetic infections of hip prostheses^56^ in patients undergoing elective hip arthroplasty; however, prophylaxis for HO after THA has not been reported. We conducted a Bayesian meta-analysis based on randomized controlled trials comparing NSAIDs with a placebo to provide a more reliable proposal for the prevention of HO after THA.

Our meta-analysis confirmed the results of Fransen and Neal^14^ in the overall incidence of HO; however, there were differences between our meta-analysis and that by Fransen and Neal.^14^ First, we excluded 2 quasi-randomized trials.^57,58^ Second, we introduced DNGSE, which was not analyzed by Fransen and Neal.^14^ By analyzing DGSE, we found that the risk of gastrointestinal side effects was increased 30.4% by NSAIDs, which was similar to the results of Fransen and Neal.^14^ However, Ma et al^53^ indicated that there was no significant difference in the gastrointestinal side effects between NSAIDs and a placebo. The number and quality of the included articles in his meta-analysis were worse than those in our meta-analysis.

In addition, a multi-center trial by Neal et al^13^ concluded that NSAIDs had no effective influence on the prevention of HO after THA. First, compared with other studies primarily examining the efficacy of NSAIDs as prophylaxis for HO after THA, this trial investigated the efficacy of low dose of NSAIDs on thrombotic and hemorrhagic outcomes rather than the efficacy on the prevention of HO after THA. This situation might introduce clinical diversity so that different results occurred. Second, the poorer efficacy of aspirin compared with other NSAIDs might induce the difference.^13^ Finally, some outcome data on HO were not available because of the drop-out rate (22.7%, 601/2649), which might also contribute to the diverse effect.

### Selective NSAIDs versus Nonselective NSAIDs

The finding was observed by comparing selective NSAIDs with nonselective NSAIDs. There was no significant difference between the selective NSAIDs and the nonselective NSAIDs in the overall incidence of HO and DNGSE. Compared with nonselective NSAIDs, selective NSAIDs yielded a significant reduction in DGSE.

No statistically significant difference in the overall incidence of HO had been indicated in individual trials.^11,37,38,51^ A similar effect on the overall incidence of HO was observed in a recent meta-analysis of randomized trials.^15^ Our meta-analysis confirmed the results of Xue et al,^15^ and because of the use of Bayesian method, our outcomes were more robust.

In addition, compared with the nonselective NSAIDs, there was a similar reduction of DGSE in selective NSAIDs between our meta-analysis and that by Xue et al.^15^ It was inferred that DGSE was the major side effect of nonselective NSAIDs, including gastric perforation, gastric ulcer, and gastrorrhagia. Bombardier et al^59^ found selective COX-2 inhibitors might have a better manifestation in the protection of gastrointestinal functioning. This finding was consistent with our outcome.

A previous study had illustrated that selective NSAIDs are less effective than nonselective NSAIDs for the prophylaxis of HO after THA.^9^ In this trial, the recruiting period was from 1996 to 1999. The time was so long that trial executors might select participants conforming to their will rather than the preplanned inclusion criteria.

As the most extensive meta-analysis, all of the available randomized controls trials of NSAIDs versus a placebo and selective NSAIDs versus nonselective NSAIDs were included, and the intrinsic risk of bias in methodology and source of data in retrospective studies was overcome. Compared with previous reviews, our sample size was reinforced, and the credibility of demonstration was enhanced. We executed this meta-analysis based on Bayesian framework, which uses the Markov chain Monte Carlo method. A Bayesian framework could calculate a relatively exact distribution of the numbered sample, directly overcoming the disadvantage of traditional methods of making a statistical inference in light of asymptotic distribution of large sample, and took into account the model's uncertainty.^16^ Therefore, our estimate outcome was more reliable and reasonable because we used the Bayesian framework.

Our meta-analysis has limitations. First, diverse dosages and types of NSAIDs were used in the 21 involved analyses. However, we did not investigate different dosages and types, because of the limited numbers of included studies, which might be a bias in the outcomes. Second, there were some patients undergoing revision surgery in the involved articles, and we could not extract the data of these patients to perform a subgroup analysis. Although there were few patients, the data might have influenced our outcomes. Finally, the statistical power in this study was slightly low, which indicated that potential differences were not identified.

## CONCLUSION

In our meta-analysis, the moderate quality of evidence indicated that NSAIDs could significantly yield a decrease in the incidence of HO, but increase DGSE. The high quality of evidence demonstrated that selective NSAIDs did not differ significantly from nonselective NSAIDs for the prevention of HO after THA, while produced fewer side effects. However, there was much indetermination in efficacy in long-range clinical outcomes. Given the side effects of nonselective NSAIDs, selective NSAIDs were recommended as prophylaxis for HO after THA. Even so, NSAIDs as prophylaxis for HO needs comprehensive evaluation in longer duration of follow-up and larger scale randomized controlled trials to identify the balance between efficacy and safety.
